# Effectiveness and Impact of the 4CMenB Vaccine against Group B Meningococcal Disease in Two Italian Regions Using Different Vaccination Schedules: A Five-Year Retrospective Observational Study (2014–2018)

**DOI:** 10.3390/vaccines8030469

**Published:** 2020-08-22

**Authors:** Chiara Azzari, Maria Moriondo, Francesco Nieddu, Valentina Guarnieri, Lorenzo Lodi, Clementina Canessa, Giuseppe Indolfi, Mattia Giovannini, Giuseppina Napoletano, Francesca Russo, Tatjana Baldovin, Silvia Cocchio, Silvia Ricci, Vincenzo Baldo

**Affiliations:** 1 Section of Pediatrics, Department of Health Sciences, University of Florence, Viale Pieraccini 24, 50139 Florence, Italy; maria.moriondo@unifi.it (M.M.); francesco.nieddu@meyer.it (F.N.); clementina.canessa@meyer.it (C.C.); mattia.giovannini@unifi.it (M.G.); 2 Immunology and Molecular Microbiology Unit, Meyer Children’s Hospital, Viale Pieraccini 24, 50139 Florence Italy; giuseppe.indolfi@unifi.it; 3 Section of Pediatrics, Department of Neurofarba, University of Florence, Viale Pieraccini 24, 50139 Florence, Italy; 4 Prevention Department, Veneto Regional Health Authority, via Don Tosatto 147, 30174 Venice Mestre, Italy; giuseppina.napoletano@ulss20.verona.it (G.N.); francesca.russo@regione.veneto.it (F.R.); 5 Department of Cardiac Thoracic Vascular Sciences and Public Health, Public Health Section, University of Padua, via Leonardo Loredan 18, 35131 Padua, Italy; tatjana.baldovin@unipd.it (T.B.); silvia.cocchio@unipd.it (S.C.); vincenzo.baldo@unipd.it (V.B.)

**Keywords:** meningococcus B, vaccination schedule, children, vaccine effectiveness, vaccine impact

## Abstract

Background: A few years after the introduction in Italy of a four-component anti-meningococcal B vaccine (4CMenB), we evaluated the effectiveness and impact of vaccination in two regions using different schedules (2, 4, 6, 12 months in Tuscany vs. 7, 9, 15 months in Veneto) through an observational retrospective study. Methods: Vaccination started in 2014 in Tuscany and in 2015 in Veneto; the data collected referred to the period 2006–2018 for Tuscany and 2007–2018 for Veneto. Cases of invasive meningococcal disease due to *N. Meningitidis* B were identified by culture and/or real-time PCR. Results: In Tuscany, pre-vaccine incidence was 1.96 (95% CL 1.52; 2.40) and dropped to 0.62 (95% CL 0.60; 0.64) in the post-4CMenB era. Evaluating only vaccinated children, post-4CMenB incidence was 0.12 (95% CL 0.08; 0.15). In Veneto pre-vaccine incidence was 1.94 (95% CL 1.92; 1.96) and dropped to 1.34 (95% CL 1.31; 1.38) in the post-4CMenB era. In the vaccinated population, MenB incidence was 0.53 (95% CL 0.50; 0.56). Vaccine effectiveness was 93.6% (95% CL 55.4; 99.1) in Tuscany and 91.0% (95% CL 59.9; 97.9) in Veneto, with mean vaccine coverages of 83.9% and 81.7%, respectively. The overall impact (evaluating both vaccinated and unvaccinated children) was 0.68 (95% CL 0.10; 0.89) in Tuscany and 0.31 (95% CL −0.56; 0.69) in Veneto; the total impact (evaluating only vaccinated children) was 0.94 (95% CL 0.56; 0.99) and 0.90 (95% CL 0.57; 0.97), respectively. The relative case reduction (RCR) was 65% in Tuscany and 31% in Veneto. Considering the vaccinated population, the RCR was equal to 91% and 80%, respectively. Conclusion: In conclusion, 4CMenB appears to have a very high effectiveness in Italy; the impact of vaccination appears greater where the immunization program is started early.

## 1. Introduction

Invasive meningococcal disease (IMD) is a severe disease mainly affecting infants and young children [1]. Most infections are caused by serogroups A, B, C, W, X, and Y. In the last 10 years, serogroup B has been the main cause of IMD in Europe and North America and one of the most prevalent serogroups in Latin America [2]. The highest incidence is found in the first year of life and in particular between the fourth and eighth months of age [3,4,5].

In 2014, the four-component anti-meningococcal B vaccine (4CMenB) vaccine (Bexsero, GSK, Rixensart, Belgium) was licensed in Italy and in 2017 it was implemented in the National Immunization Program as four-dose schedule starting at 2 months of age [6]. However, already in 2014 and 2015, respectively, two Italian Regions introduced into their routine a publicly funded infant immunization program. Two different schedules were used: Tuscany offered 4CMenB as a four-dose schedule (2, 4, 6, and 12–13 months of age), while Veneto as a three-dose schedule (7, 9 and 15 months of age).

Since 4CMenB has been licensed on the basis of safety and immunogenicity studies without real-world evidence of effectiveness, collecting data in observational studies from real-world usage is becoming crucial to confirm the ability of the vaccine to protect against IMD caused by serogroup B.

The aim of the present work was to evaluate the effectiveness and impact of the 4CMenB vaccine in two Italian Regions and to compare two different vaccination schedules currently in use.

## 2. Materials and Methods

### 2.1. Study Setting

Tuscany is a region in the center of Italy and has a population of 3.7 million inhabitants, with an average number of 27,552 births per year in the last 5 years. The Tuscany region has a structured network for molecular surveillance of vaccine preventable diseases. It was started in 2005, in addition to standard culture surveillance, based in the Laboratory of Immunology and Molecular Microbiology at the Meyer Children’s University Hospital in Florence, which was chosen as the reference center for meningococcal, pneumococcal, and *Haemophilus influenzae* diseases by a regional law (DGR 571/2015). The Laboratory is located at the III level hospital to which all pediatric cases of IMD from Tuscany region are referred. Therefore, missing cases are extremely unlikely. Case ascertainment and follow-up is performed through clinical, public health and laboratory data.

Veneto is a region in northeast Italy with a population of 4.9 million inhabitants and an average of 38,992 births per year in the last 5 years. Diagnosis and grouping of IMD are made either by culture or by molecular methods. In 2007 an active surveillance system of invasive bacterial diseases was implemented. All suspected clinical cases of IMD are reported by the laboratories to the Regional Epidemiology Centre and concurrently biological samples are sent to the Regional Reference Laboratory for culture confirmation and serotyping of the isolates; all data are routinely merged by Public Health Surveillance [7].

### 2.2. Vaccination Schedules and Coverage

Tuscany and Veneto introduced 4CMenB in their vaccination program anticipating the National Immunization Program (2017–2019) indications.

In Tuscany, vaccination was offered to all infants born from January 2014 on a four-dose schedule (2, 4, 6 and 12–13 months of age). In Veneto vaccination was started in 2015 on a three-dose schedule (7, 9 and 15 months of age). No catch-up campaign for children > 1 year of age was planned in the two regions.

Vaccination coverage was obtained from an integrated regional immunisation registry linked to the population registry and was calculated as cohort coverage at 24th month for the cohorts 2014–2017 in Tuscany and 2015–2017 in Veneto (Table 1).

### 2.3. Study Design

Our observational study evaluated retrospectively the incidence of IMD due to *Neisseria meningitidis* group B in the pre-vaccination era (pre-4CMenB) and in the post-vaccination era (post-4CMenB) in two Italian regions using two different vaccination protocols.

Since vaccination was introduced in Tuscany in 2014 and in Veneto in 2015, the post-vaccine era was defined as 2014–2018 in Tuscany and 2015–2018 in Veneto. Therefore, data referred to children 0–5 years of age in Tuscany and 0–4 years of age in Veneto.

For the pre-vaccine era, given the rarity of meningococcal disease, in order not to miss any available information, we evaluated all data provided by regional registers [8]. Thus, in the Tuscany pre-vaccination period was defined as 2006–2013 and in Veneto as 2007–2014.

Clinical and laboratory data were recorded using a standardized report form.

All data and samples were collected as part of the routine clinical activity and the vaccine preventable disease surveillance activity required by law and were evaluated retrospectively and anonymously in the study. The study was approved by the Pediatric Regional Ethical Committee.

### 2.4. Case Definition

A diagnosis of laboratory-confirmed IMD was made if a patient’s samples were culture positive for *Neisseria meningitidis*, or real-time PCR positive for the ctrA gene, or both, as described previously [4]. Serogroup B [9] was identified by real-time PCR as previously described [8].

IMD cases which resulted up to date with their regional vaccination schedule were considered as vaccinated, regardless the number of doses received (at least one).

### 2.5. Laboratory Methods

RT-PCR and culture methods have been previously described [8,10]. In brief, for culture purposes, standardized procedures were used for collection and shipment of biological samples from the clinic to local laboratories (Laboratory of Immunology and Molecular Microbiology at the Meyer Children’s University Hospital in Florence, Tuscany; regional reference laboratories accredited to Italian National Healthcare System in Veneto). Real-time PCR reliability in meningococcal diagnosis and serogrouping had been previously demonstrated by testing inactivated meningococcal serogrouped isolates from ATCC as controls [10].

### 2.6. Evaluation of Vaccine Effectiveness

Vaccine effectiveness (VE) was calculated using the Farrington screening method [11], according to Parikh SR et al. [12] with the following formula:

PCV
VE = 1 1 − PCV
PPV
1 − PPV

where PCV is the proportion of children vaccinated with 4CMenB among IMD cases and PPV is the mean vaccine coverage in age-matched children. An estimated effectiveness for the entire study population (0–5 years for Tuscany and 0–4 for Veneto) was calculated assuming PPV as the mean of the cohorts’ vaccine coverages for each region (Table 1).

### 2.7. Evaluation of Incidence and Impact of Vaccination

The IMD incidence rates (number of cases on 100,000 children) were calculated as crude incidence rates in the pre-vaccine era (reference population) and in the post-vaccine era (study population). Furthermore, an Age Standardized Incidence Rate (ASR) was calculated in the post-vaccine era, weighted on the distribution of incidence rate of the reference population. The ASR was necessary to eliminate the difference in age distribution among reference and study populations.

The impact of 4CMenB vaccination was estimated considering the reduction of incidence rates by comparing pre- and post-vaccine incidence rates for each age group with the following formula:

Impact = 1 − IRR

Incidence Rate Ratio (IRR) = Incidence rate in post-vaccine era/Incidence rate in pre-vaccine era

For the assessment of impact on each age group, pre- and post-vaccine crude incidences were used. For evaluation of the impact on the entire population, ASR was used for the post-vaccine era.

We also used, as an additional impact indicator, the reduction of risk of disease expressed by the number of cases prevented in the post-4CMenB era (Relative Case Reduction, RCR). It was calculated as follows:

Relative Case Reduction (RCR) = 1 − SIR

Standardized Incidence Ratio (SIR) = Observed Cases/Expected Cases

Expected cases were estimated on the basis of the pre-vaccination incidence rate for each age class. The vaccine impact was evaluated as overall impact (the effect of the 4CMenB vaccination program on the entire population, regardless of vaccination status) and the total impact (the effect of 4CMenB vaccination on the vaccinated population).

### 2.8. Statistical Analysis

The data were processed with the SPSS release 21 statistical package and the freely available “epitools” R package (https://www.r-project.org/). *p* < 0.05 was considered to be statistically significant and 95% confidence intervals were shown when appropriate. Odd ratios were evaluated using Java-Stat free. Confidence intervals for effectiveness and for impact were calculated with the Wilson method.

## 3. Results

### 3.1. Incidence of Invasive Meningococcal Disease in Pre- and Post 4CMenB Era

**Figure 1:**
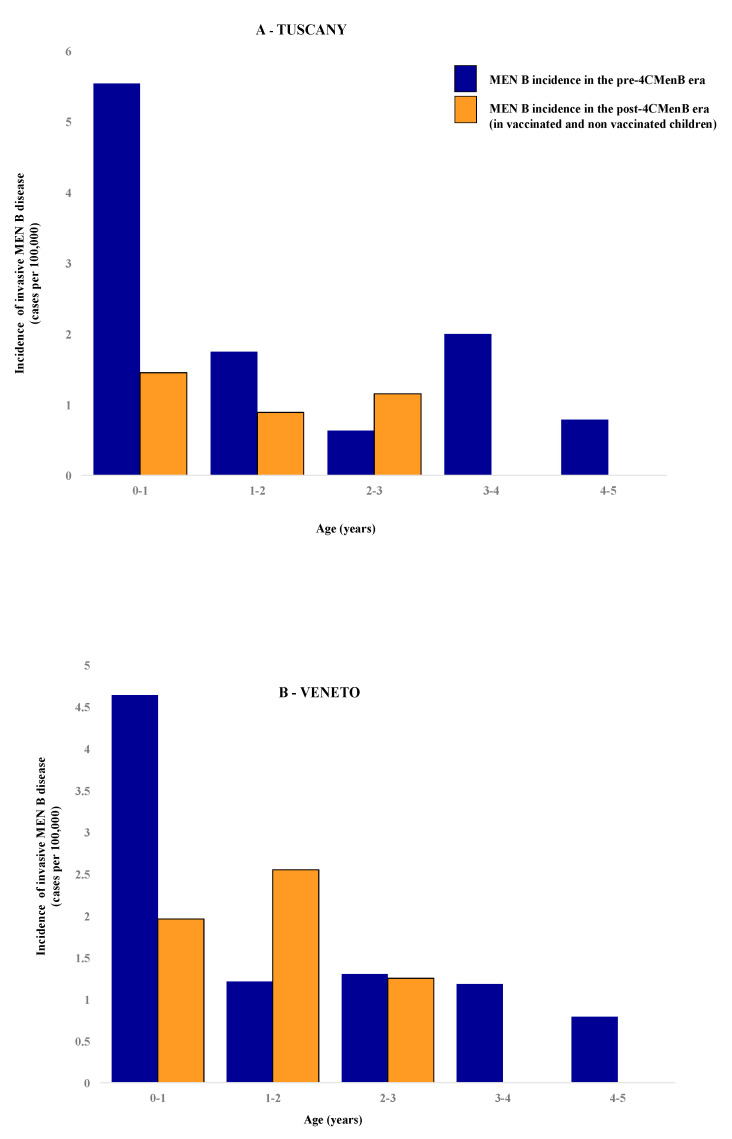
Crude incidences of invasive meningococcal disease in children 0–5 years of age pre and post introduction of 4CMenB in Tuscany (**A**) or Veneto (**B**). In Tuscany, 3 out of 4 cases of Invasive Meningococcal Disease (IMD) and in Veneto 5 out of 7 cases of IMD in the post-4CMenB era occurred in not-vaccinated children. Abbreviations: MenB: *Neisseria meningitides* serogroup B; 4CMenB = multicomponent meningococcal serogroup B vaccine.

Cases of IMD due to *Neisseria meningitidis* B (MenB) according to age are shown in Table 2; population distribution over time in the two regions is shown in the Supplementary File (Figure S1A,B). Descriptive data (crude incidences, ASR, IRR, Impact, RCR and SIR) in vaccinated and non-vaccinated children are presented in Table 3 and Table 4 and in Figure 1.

In Tuscany, 31 cases of MenB were diagnosed in children 0–5 years old during the pre-4CMenB era and four cases in the post-vaccine era; in Veneto, 34 cases in the pre-4CMenB and seven cases in the post vaccine era were identified in children 0–4 years of age. The crude incidence of MenB in Tuscany was 1.96 (95% CL 1.52; 2.40) per 100,000 children in the pre-vaccine era and it dropped to an ASR of 0.62 (95% CL 0.60; 0.64; *p* = 0.058; OR 3.14, 95% CL 0.895; 11.004) in the post-4CMenB era. In Veneto, it was 1.94 (95% CL 1.92; 1.96) in the pre-vaccine era while the ASR in the post-vaccine era was 1.34 (95% CL 1.31; 1.38, *p* = ns, OR 1.44, 95% CL 0.58; 3.63).

With respect to age distribution, the highest number of cases was found in the age group 0–1 year: 14 cases were found in Tuscany (14/31, 45.2%) and 17 cases (17/34, 50%) in Veneto in the pre-4CMenB era. As for the post-vaccine era, two out of four MenB cases occurred in infants 0–1 year of age in Tuscany (2/4, 50%) and three out of seven (3/7, 42.9%) in Veneto. In Tuscany the crude pre-vaccine incidence rate was 5.54 per 100,000 and dropped to 1.45 (*p* = 0.067; OR = 3.81; 95% CL 0.87; 16.78) in the post-vaccine era. Similarly, in Veneto, the pre-vaccine incidence was 4.65 (95% CL 4.33; 4.97) per 100,000 infants 0–1 years of age and dropped to 1.96 (95% CL 1.52; 2.40) in post-vaccine era (*p* = 0.220; OR 2.38, 95% CL 0.70; 8.11).

With respect to vaccination status, in Tuscany, among four cases found in post-vaccine era, one occurred in an infant vaccinated with two doses of 4CMenB (7 months of age) and three cases were found in non-vaccinated children (respectively, in the first, second and third year of life). In Veneto, among seven cases found in the post-4CMenB era, two cases occurred in vaccinated children (1–2 age groups) and five cases in unvaccinated children (three in 0–1 age group, one in 1–2 and one in 2–3 year classes). More specifically, in Veneto, where the vaccination schedule starts at the 7th month of age, three unvaccinated cases occurred at 1, 3 and 4 months of age (the latter died due to fulminant IMD). When only vaccinated children were evaluated, in Tuscany, the crude incidence in the post-vaccine era was 0.73 per 100,000 (95% CL 0.65; 0.81), the ASR was 0.12 (95% CL 0.08; 0.15; *p* = 0.003; OR 16.6; 95% CL 1.02; 271.95); in Veneto the crude incidence was 0.85 (95% CL 0.56; 1.14) and the ASR was 0.20 (95% CL 0.06; 0.34; *p* = 0.007; OR 9.50; 95% CL 1.03; 87.22).

### 3.2. Effectiveness of Vaccination

Among MenB cases registered during the post-vaccine period, the proportion of vaccinated children (PCV) in Tuscany was equal to 25% (one vaccinated child out of four total cases), the mean vaccine coverage (PPV) was equal to 83.9%. In Veneto, the PCV was equal to 28.6% (two out of seven total cases) and PPV to 81.7%.

Thus, considering the entire population of study, the estimated vaccine effectiveness was 93.6% (95% CL 55.4; 99.1) in Tuscany and 91.0% (95% CL 59.9; 97.9) in Veneto.

As for MenB cases registered in each age class, we recorded one vaccinated out of two total cases 0–1 years of age in Tuscany (PCV = 50%) and two vaccinated out of three total cases in class 1–2 years of age in Veneto (PCV = 66%). Given a vaccine age-matched coverage (PPV) of 84.9% for children 0–1 in Tuscany and a mean PPV of 80.3% (mean of cohorts 2015 and 2016) for children 1–2 years of age in Veneto, vaccine effectiveness for the 0–1 years of age group resulted 82.2% (95% CL −70.3; 98.1) in Tuscany and for 1–2 years of age group was 50.9% (95% CL −274.4; 93.5) in Veneto (Table 1). For both regions, PCV values calculated in the other classes of age were equal to zero.

### 3.3. Impact of Vaccination in Tuscany and Veneto

**Figure 2:**
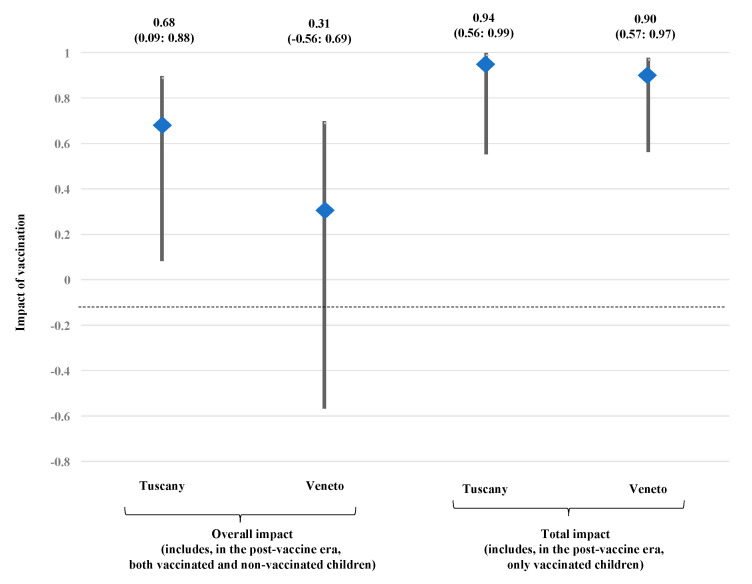
Impact of vaccination schedule in Tuscany and Veneto. Impact was evaluated as overall impact (including, in the post-4CMenB era, both vaccinated and not vaccinated children) and as total impact (including, in the post-4CMenB era, only vaccinated children).

The incidence rate ratio (IRR) and the impact of vaccination in the two regions are shown in Table 3. The overall impact, evaluated including cases recorded in both vaccinated and non-vaccinated children, was 0.68 (95% CL 0.09; 0.88 in Tuscany and 0.31 (−0.56; 0.69) in Veneto. The total impact, including cases found in vaccinated children only was 0.94 (95% CL 0.56; 0.99) in Tuscany and 0.90 (95% CL 0.57; 0.97) in Veneto. The data are shown in Figure 2.

As for the first year of life—the age with the highest incidence in both regions—the overall impact was 0.74 (95% CL −0.15; 0.94) in Tuscany and 0.58 (−0.43; 0.87) in Veneto.

In the post-vaccine era, four cases were observed versus 11.6 cases expected in Tuscany (Table 4). The SIR was 0.35 and the relative case reduction (RCR) was 65.4%. Considering the vaccinated population only, the SIR was 0.09 and the RCR reached was 91.3%.

In the class with the highest pre-vaccine incidence, the 0–1 years of age class, the RCR was 74% (SIR = 0.26) when both vaccinated and unvaccinated children were evaluated and 87% (SIR = 0.13) for vaccinated children.

In Veneto, seven cases were observed versus the 10.2 cases expected. The SIR was 0.69 with an RCR of 31.2%. Considering the only vaccinated population (0–4 years old), the RCR reached 80.3% (SIR = 0.20). In age group 0–1, the SIR was 0.42 with an RCR of 58%.

## 4. Discussion

The study demonstrates that the anti-meningococcus B vaccine, 4CMenB, has an effectiveness of 91–93.6% in preventing IMD due to serogroup B in two Italian regions where the vaccination has been included in the Regional Immunization Program for infancy.

The results confirm data obtained by Parikh et al. and Ladhani et al. [12,13] in the UK.

In Tuscany, where vaccination with 4CMenB was offered free of charge to all children in the first year of life on a 4-dose schedule (2, 4, 6, 12–13 months), in the post vaccination era, the incidence rate in children 0–5 years (including both vaccinated and unvaccinated children) decreased from 1.96 to 0.62 per 100,000 children with an overall impact of 68%. The reduction was even more pronounced in the class age with the highest incidence of risk, that of children 0–1 year of age. In that cohort, the incidence decreased by 74% from 5.54 to 1.45 per 100,000 children.

In Veneto, where the vaccination program started 1 year later with a 3-dose schedule starting in the second semester of life (7, 9, 15 months), the incidence in children 0–4 years decreased from 1.94 to 1.34 per 100,000 with an overall impact of 31%; in the cohort of children 0–1 years of age, it decreased by 58% from 4.65 to 1.96 per 100,000.

The trends in IMD cases due to *Neisseria meningitidis* B demonstrated a rapid decrease in both Italian regions, with an almost complete disappearance of cases in vaccinated children (total impact of 94% in Tuscany and 90% in Veneto) that was already evident in the first year after the start of the vaccination program. These data confirm what was demonstrated in the UK 10 months after the introduction of vaccination [12].

The higher impact observed in Tuscany respect to Veneto is probably due to the earlier vaccine administration starting from the second month of life. That difference was not completely unexpected since in Italy [4], as in the UK [5], the peak of incidence for meningococcal disease in the first year of age is between the fourth and the eighth month of age [4].

As a confirmation of this, three cases of IMD due to MenB occurred in non-vaccinated children in the first year of life in the Veneto region: one child aged one month, one child aged three months and one child aged four months (the latter child died due to fulminant IMD). With the vaccination schedule being used in Tuscany, two out of three of those children would have received at least the first dose of vaccine. That dose might be useful to prevent IMD: recent data from Parikh et al. [12] on children vaccinated with a single dose of 4CMenB suggested a vaccine effectiveness of 64.0% (95% CI 8.9; 84.0).

Data obtained in the UK [13] demonstrated that a 3-dose schedule started in the second month of life has a good effectiveness and a significant impact on the population of vaccinated children, and it has been suggested that starting the vaccination early is more important than the number of doses.

Our data also suggest that protection induced by vaccination persists in the following years. Actually, the three IMD cases that occurred in vaccinated children were found within one year from vaccination, suggesting that vaccine failure was probably due to an incomplete coverage of 4CMenB against circulating strains, more than to antibody decay over time in the host. 4CMenB is a multi-component vaccine containing three surface-exposed recombinant proteins (fHbp, NadA, and NHBA) and PorA 1.4 [14]. The effectiveness of the vaccination is known to depend on the presence, in circulating strains of meningococcus B, of the antigens included in the vaccine. Therefore, the effectiveness of the vaccine may vary in different geographical areas. However, results obtained up to now in different countries of the world, from Italy to the UK to Canada [12,15,16], demonstrate a significantly high effectiveness of the vaccine, greater than that predicted by the Meningococcal Antigen Typing System in pre-marketing studies [17].

Molecular studies are now ongoing in Italy, carried out by the Laboratory of the Meyer Hospital in Tuscany, to evaluate the distribution of subcapsular proteins in IMD cases due to meningococcus B and possible variations of the subcapsular protein expression facilitated by vaccine pressure.

This study has a weakness, due to the fact that confidence intervals for effectiveness and impact are large because of the small numbers of IMD cases found in the two regions during the follow-up.

On the other hand, the inclusion of the two regions considered, Tuscany and Veneto, is a strength of the study since in both regions an enhanced surveillance program is in force and molecular biology for diagnosis and serogrouping/serotyping of IMD has been in place for many years [4,8,18].

Further studies, including more Italian regions, are currently in progress in Italy, and these will allow the effectiveness and impact of 4CMenB to be confirmed and the molecular vaccine coverage to be monitored.

## 5. Conclusions

The present study shows a significant reduction in the number of IMD cases due to *Neisseria meningitidis* B after the introduction of 4CMenB in two different Italian regions, demonstrating the consistent impact of the vaccination program in infants. The estimated effectiveness was high in both regions. These data support the prevention potential of a 4CMenB vaccination program on a national scale.

Moreover, the different schedule implemented in the two regions represented a unique opportunity for comparison of vaccine administration timing. Our results suggest that the early start of vaccination (2 months of life) seems preferable compared to a later start (7 months of life). In fact, the vaccination impact observed is higher when vaccination is started earlier. The early-starting strategy ensured a greater level of protection of children between the fourth and the eight months of life, which represents the age group at the highest risk of IMD due to *Neisseria meningitidis* B.

## Figures and Tables

**Table 1:** 4CMenB coverage in Tuscany and Veneto during the post-vaccination era (cohort at 24th months).

Cohort Group by Age of Birth	Coverage % at 24 Months Per Year	
	TUSCANY	VENETO
2017	84.9 (2019)	84.7 (2019)
2016	86.1 (2018)	82.0 (2018)
2015	88.1 (2017)	78.6 (2017)
2014	76.8 (2016)	n.a.
Mean coverage	83.9	81.7

Abbreviations: n.a. = not available; % = percentage.

**Table 2:** Distribution of *Neisseria meningitidis* B cases based on age groups and year of diagnosis in Tuscany (2006–2018) and Veneto (2007–2018).

**(A) TUSCANY**													
	**2006**	**2007**	**2008**	**2009**	**2010**	**2011**	**2012**	**2013**	**2014**	**2015**	**2016**	**2017**	**2018**
**Age class**													
**4–5**	**1**				**1**					**1**			
**3–4**				**1**	**1**		**1**	**2**	**1**	**1**			
**2–3**				**1**			**1**						**1**
**1–2**				**2**	**1**	**1**	**0**		**1**			**1**	
**0–1**	**2**	**1**	**3**	**1**	**2**	**1**	**2**	**2**					**2**
**TOTAL**	**3**	**1**	**3**	**5**	**5**	**2**	**4**	**4**	**2**	**2**	**0**	**1**	**3**
**(B) VENETO**													
		**2007**	**2008**	**2009**	**2010**	**2011**	**2012**	**2013**	**2014**	**2015**	**2016**	**2017**	**2018**
**Age class**													
**3–4**		**1**				**1**	**1**	**1**	**1**	**1**			
**2–3**		**1**	**1**			**2**	**1**					**1**	
**1–2**		**1**	**1**		**1**	**2**		**1**				**1**	**2**
**0–1**		**1**	**3**	**2**	**3**	**1**	**3**	**3**	**1**	**1**		**1**	**1**
**TOTAL**		**4**	**5**	**2**	**4**	**6**	**5**	**5**	**2**	**2**	**0**	**3**	**3**

Areas highlighted in gray represent the vaccine-eligible cohorts since 2014 in Tuscany and 2015 in Veneto.

**Table 3:** *Neisseria meningitidis* B cases, incidences and impact for age groups, vaccinated or not, in Tuscany and Veneto during pre and post-vaccination eras.

	PRE—4CMenB		POST—4CMenB									
Age Groups	Number of Cases	Incidence * (Cases/100.000)	Number of Cases	Vaccinated Cases	Crude Incidence * (Cases/100.000)	ASR	Crude Incidence on Vaccinated Cases	ASR on Vaccinated Cases	IRR	IRR on Vaccinated Cases	Overall Impact (%)	Total Impact (%)
**(A) TUSCANY**												
0–1	14	5.537 (5.44–5.62)	2	1	1.452 (1.39–1.51)	0.232	0.726	0.116	0.260	0.131	74%	87%
1–2	5	1.749 (1.70–1.80)	1		0.887 (0.68–1.02)	0.160			0.507		49%	
2–3	2	0.631 (0.60–0.66)	1		1.160 (1.00–1.50)	0.232			1.838			
3–4	7	2.007 (1.96–2.05)	0									
4–5	3	0.789 (0.76–0.82)	0									
**TOTAL**	**31**	**1.956**	**4**	**1**	**0.941**	**0.624**	**0.726**	**0.116**	**0.320**	**0.060**	**68%**	**94%**
**(B) VENETO**												
0–1	17	4.647 (4.33–4.97)	3		1.956 (1.52–2.40)	0.409			0.421		58%	
1–2	6	1.447 (1.41–1.48)	3	2	2.552 (2.46–2.64)	0.605	0.851	0.200	1.764	0.590		41%
2–3	5	1.082 (1.05–1.11)	1		1.253 (1.18–1.33)	0.330			1.158			
3–4	6	1.181 (1.15–1.21)	0									
**TOTAL**	**34**	**1.942**	**7**	**2**	**1.789**	**1.344**	**0.851**	**0.200**	**0.692**	**0.103**	**31%**	**90%**

Abbreviations: 4CMenB = multicomponent meningococcal serogroup B vaccine; ASR = Age Standardized Incidence; Rate IRR = Incidence Rate Ratio. * in brackets 95% CL.

**Table 4:** Standardized Incidence Ratio and Relative Case Reduction in *Neisseria meningitidis* B cases (vaccinated or not) in Tuscany and Veneto.

Age Groups	Observed Cases	Vaccinated Cases	Expected Cases	SIR	SIR on Vaccinated Population	RCR	RCR on Vaccinated Population
**(A) TUSCANY**							
0–1	2	1	7.628	0.262	0.131	0.738	0.869
1–2	1	-	1.972	0.507	-	0.493	-
2–3	1	-	0.544	1.838	-	0.838	-
3–4	0	-	1.180	0	-	1	-
4–5	0	-	0.234	0	-	1	-
**TOTAL**	**4**	**1**	**11.558**	**0.346**	**0.087**	**0.654**	**0.913**
**(B) VENETO**							
0–1	3	-	7.128	0.421	-	0.579	-
1–2	3	2	1.701	1.764	1.176	−0.764	−0.176
2–3	1	-	0.864	1.158	-	−0.158	
3–4	0	-	0.480	0	-	1	
**TOTAL**	**7**	**2**	**10.173**	**0.688**	**0.197**	**0.312**	**0.803**

Abbreviations: RCR = Relative Case Reduction; SIR = Standardized Incidence Ratio.

## Supplementary Materials

The following are available online at https://www.mdpi.com/2076-393X/8/3/469/s1, Supplementary Figure S1A: Evolution of pediatric population for age groups in Tuscany (0–5 years in 2006–2018, ISTAT). Supplementary Figure S1B: Evolution of pediatric population for age groups in Veneto (0–4 years in 2007–2018, ISTAT).
