# Supplementary figures and images for: Effectiveness and Impact of the 4CMenB Vaccine against Group B Meningococcal Disease in Two Italian Regions Using Different Vaccination Schedules: A Five-Year Retrospective Observational Study (2014–2018)
Source: Vaccines (Basel). 2020 Aug 22;8(3):469. doi: 10.3390/vaccines8030469 (PMC7563708; doi:10.3390/vaccines8030469)

## Slide 1
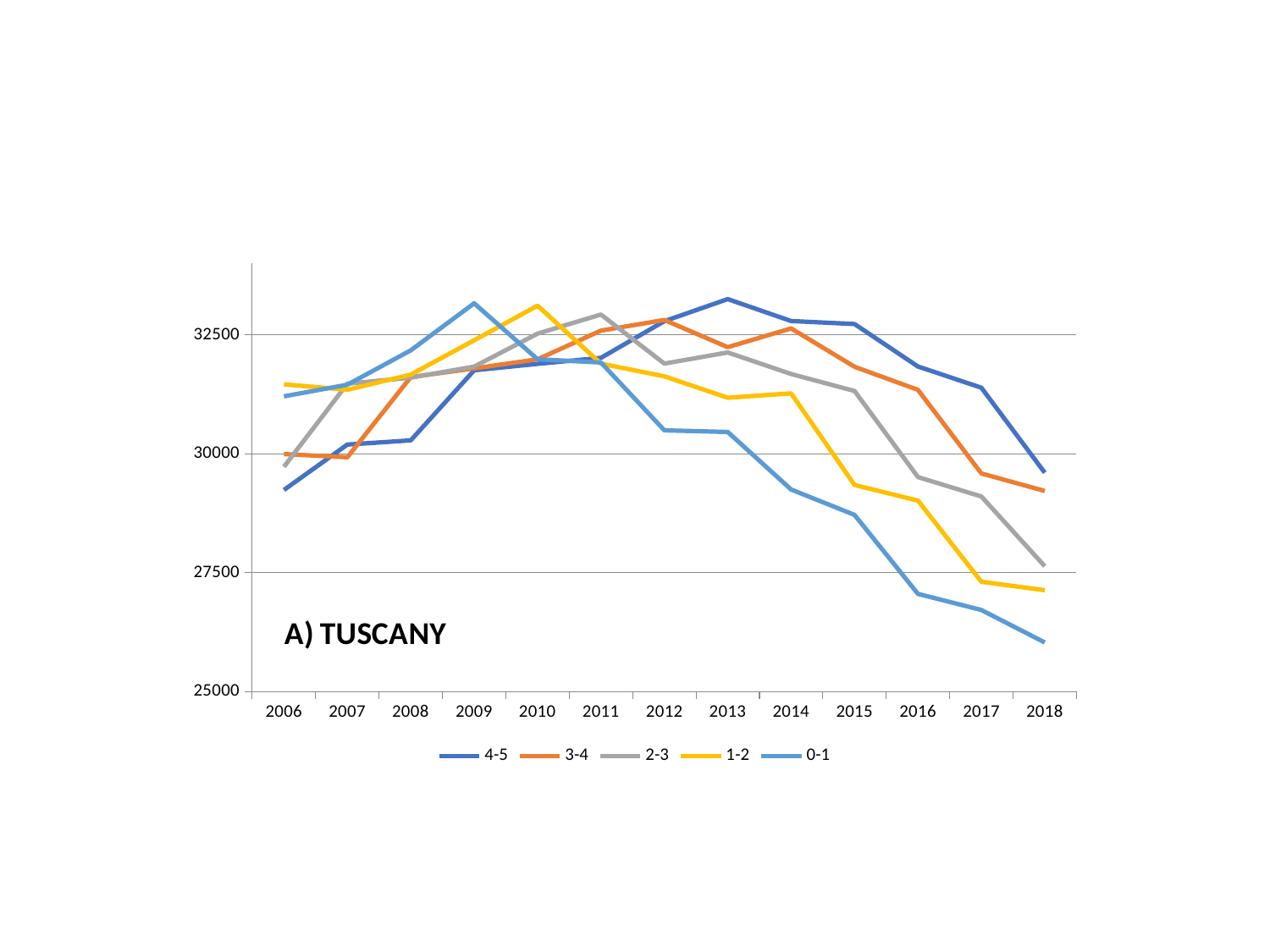

### Chart: A) TUSCANY
| Category | 4-5 | 3-4 | 2-3 | 1-2 | 0-1 |
|---|---|---|---|---|---|
| 2006.0 | 29237.0 | 29993.0 | 29726.0 | 31459.0 | 31206.0 |
| 2007.0 | 30192.0 | 29925.0 | 31471.0 | 31343.0 | 31450.0 |
| 2008.0 | 30280.0 | 31610.0 | 31599.0 | 31663.0 | 32172.0 |
| 2009.0 | 31753.0 | 31793.0 | 31831.0 | 32383.0 | 33161.0 |
| 2010.0 | 31888.0 | 31984.0 | 32524.0 | 33111.0 | 31985.0 |
| 2011.0 | 32014.0 | 32587.0 | 32925.0 | 31893.0 | 31917.0 |
| 2012.0 | 32785.0 | 32811.0 | 31894.0 | 31627.0 | 30491.0 |
| 2013.0 | 33250.0 | 32239.0 | 32128.0 | 31176.0 | 30457.0 |
| 2014.0 | 32788.0 | 32633.0 | 31675.0 | 31268.0 | 29246.0 |
| 2015.0 | 32725.0 | 31827.0 | 31318.0 | 29341.0 | 28713.0 |
| 2016.0 | 31833.0 | 31342.0 | 29508.0 | 29014.0 | 27053.0 |
| 2017.0 | 31387.0 | 29585.0 | 29100.0 | 27308.0 | 26715.0 |
| 2018.0 | 29601.0 | 29216.0 | 27635.0 | 27130.0 | 26032.0 |

## Slide 2
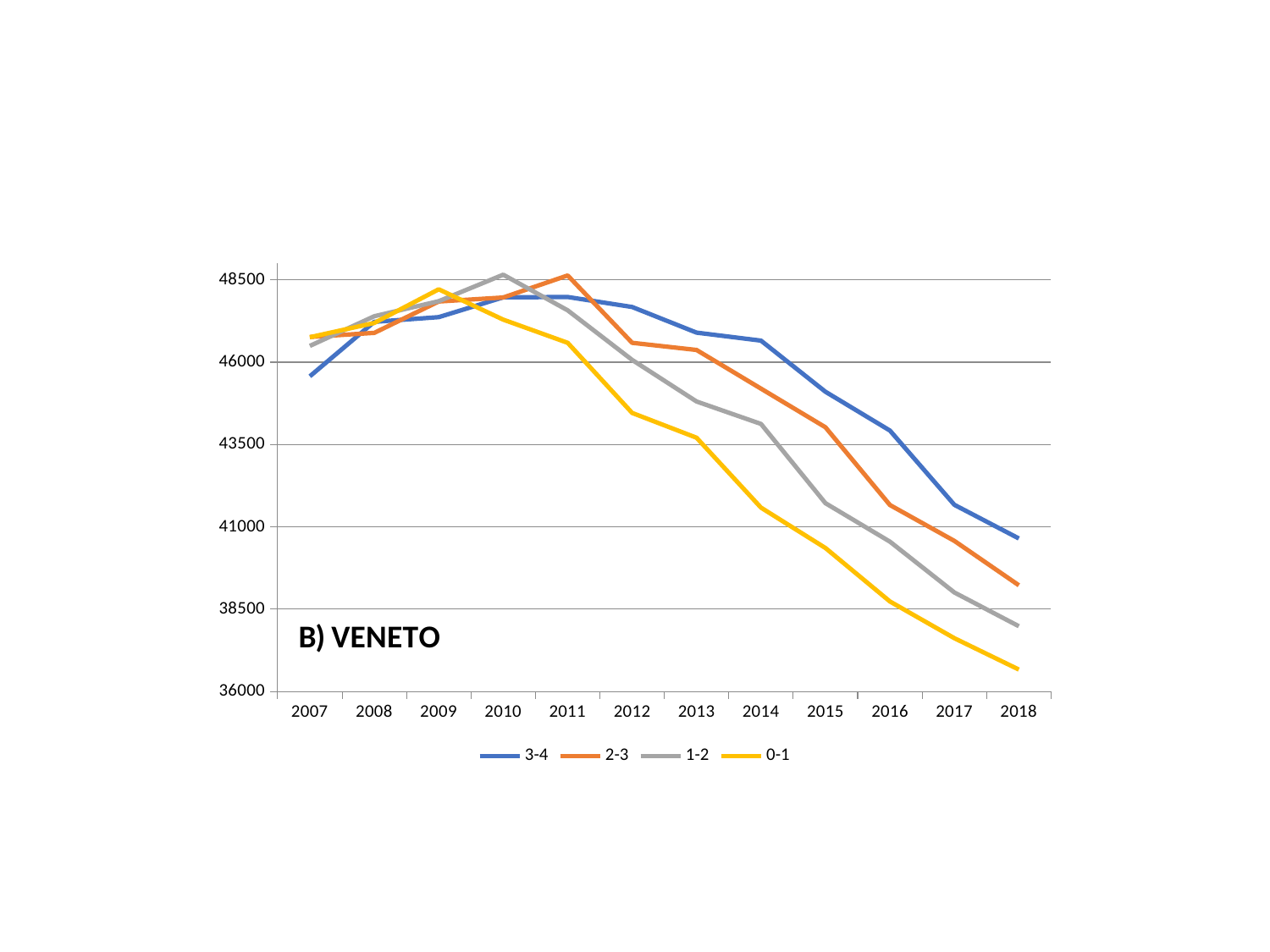

### Chart: B) VENETO
| Category | 3-4 | 2-3 | 1-2 | 0-1 |
|---|---|---|---|---|
| 2007.0 | 45572.0 | 46766.0 | 46494.0 | 46759.0 |
| 2008.0 | 47225.0 | 46891.0 | 47395.0 | 47191.0 |
| 2009.0 | 47369.0 | 47843.0 | 47852.0 | 48214.0 |
| 2010.0 | 47969.0 | 47964.0 | 48656.0 | 47293.0 |
| 2011.0 | 47979.0 | 48634.0 | 47574.0 | 46588.0 |
| 2012.0 | 47677.0 | 46589.0 | 46070.0 | 44460.0 |
| 2013.0 | 46898.0 | 46372.0 | 44808.0 | 43708.0 |
| 2014.0 | 46656.0 | 45198.0 | 44127.0 | 41583.0 |
| 2015.0 | 45103.0 | 44024.0 | 41719.0 | 40358.0 |
| 2016.0 | 43924.0 | 41663.0 | 40550.0 | 38734.0 |
| 2017.0 | 41669.0 | 40573.0 | 39007.0 | 37620.0 |
| 2018.0 | 40647.0 | 39226.0 | 37984.0 | 36668.0 |

Supplement: Supplementary file 1 [file vaccines-08-00469-s001.zip › vaccines-891647-supplementary.pptx]
